# An Overview of the Use of Genotyping Techniques for Assessing Genetic Diversity in Local Farm Animal Breeds

**DOI:** 10.3390/ani11072016

**Published:** 2021-07-06

**Authors:** Anna Olschewsky, Dirk Hinrichs

**Affiliations:** Animal Breeding Section, University of Kassel, 37213 Witzenhausen, Germany; dhinrichs@agrar.uni-kassel.de

**Keywords:** genetic diversity, local farm animal breeds, genomic techniques, conservation

## Abstract

**Simple Summary:**

The number of local farm animal breeds is declining worldwide. However, these breeds have different degrees of genetic diversity. Measuring genetic diversity is important for the development of conservation strategies and, therefore, various genomic analysis techniques are available. The aim of the present work was to shed light on the use of these techniques in diversity studies of local breeds. In summary, a total of 133 worldwide studies that examined genetic diversity in local cattle, sheep, goat, chicken and pig breeds were reviewed. The results show that over time, almost all available genomic techniques were used and various diversity parameters were calculated. Therefore, the present results provide a comprehensive overview of the application of these techniques in the field of local breeds. This can provide helpful insights into the advancement of the conservation of breeds with high genetic diversity.

**Abstract:**

Globally, many local farm animal breeds are threatened with extinction. However, these breeds contribute to the high amount of genetic diversity required to combat unforeseen future challenges of livestock production systems. To assess genetic diversity, various genotyping techniques have been developed. Based on the respective genomic information, different parameters, e.g., heterozygosity, allele frequencies and inbreeding coefficient, can be measured in order to reveal genetic diversity between and within breeds. The aim of the present work was to shed light on the use of genotyping techniques in the field of local farm animal breeds. Therefore, a total of 133 studies across the world that examined genetic diversity in local cattle, sheep, goat, chicken and pig breeds were reviewed. The results show that diversity of cattle was most often investigated with microsatellite use as the main technique. Furthermore, a large variety of diversity parameters that were calculated with different programs were identified. For 15% of the included studies, the used genotypes are publicly available, and, in 6%, phenotypes were recorded. In conclusion, the present results provide a comprehensive overview of the application of genotyping techniques in the field of local breeds. This can provide helpful insights to advance the conservation of breeds.

## 1. Introduction

According to the FAO [1], the number of farm animal breeds is declining, predominantly due to the intensification of farming, which has been happening since the middle of the 20th century. Among other factors, this is related to increasing areas of monoculture, a decoupling of livestock production and the worldwide use of a few high performing livestock breeds [1]. The FAO [1] reported that 24% of almost 9000 farm animal breeds are at risk, while the risk status of 59% is unknown. Furthermore, 7% are considered extinct, while only 10% of farm animal breeds have been classified as not at risk [1].

The majority of farm animal breeds (88%) are classified as “local breeds” [1]. This means that they are adapted to local conditions and a particular production environment [1]. Altogether, these local breeds provide a high amount of genetic diversity [1], particularly in developed regions [2]. The FAO [3] further stated that isolated and well-defined populations may have a lower extent of genetic diversity, whereas less-defined breeds tend to have a higher genetic diversity. Additionally, breeds that are shaped by local selection through farmers themselves tend to have a higher amount of diversity and are less genetically uniform compared to breeds from breeding companies [1]. Thus, local breeds exhibit varying degrees of genetic diversity, which makes it important to measure diversity in these breeds for prioritization and conservation decision making [1,4]. Conserving breeds provides insurance against future challenges such as the long-term effects of climate change or the emergence of new and unknown diseases [1].

Phenotype and pedigree information were traditionally used to describe a breed or identify members of a breed. Today, there are a number of marker-based techniques that are useful to analyze the genetic diversity of breeds [5,6]. Yaro et al. [6] distinguished between seven different types of marker techniques that are predominantly used in animal diversity studies. These include restriction fragment length polymorphism (RFLP), mitochondrial DNA barcoding (mtDNA), random amplified polymorphic DNA (RAPD), the amplified fragment length polymorphism technique (AFLP), the y-chromosome technique, the use of a variable number of tandem repeats (VNTR) including minisatellite and microsatellite markers, and single nucleotide polymorphism (SNP).

The use of microsatellite markers started in the 1990s and offered a number of advantages that go beyond diversity studies; however, these have been largely replaced by the increased use of SNP-arrays [5,6]. Such SNP-arrays provides a higher marker density and, therefore, extensive information about the genetics of an animal can be obtained [5,6]. The development of SNP-arrays is based on selected breeds of a species and is, therefore, not always representative for every population. In particular, some ascertainment bias may arise when genotyping local breeds, which may have clearly deviating traits [5,6,7]. However, further progress in the development of SNP-arrays and the use of sequencing has overcome this problem. For example, the use of high-density arrays contributed to an increased clarification of the genome. Furthermore, the advent of second and third generation sequencing resulted in a reduction in costs and a subsequent introduction of whole-genome sequencing (WGS), especially since 2007. The deployment of SNP technology allows for the amassment of detailed information on rare breeds with special genetic structure [5,6].

Several authors [8,9], have highlighted the crucial need to control inbreeding and coancestry when maintaining genetic diversity. Furthermore, both within- and between-breed genetic diversity should be considered in breed or species conservation programs [9]. These two components constitute total genetic diversity and can be measured in terms of different parameters [9]. With the assessment of genetic distances, Weitzman [10] has laid a foundation that sheds light on between-breed diversity. Between-breed genetic differentiation can be further quantified and visualized by phylogenetic reconstruction [9,11]. Genetic diversity within a breed is mostly described by assessing heterozygosity [5,9,11,12]. Cluster analyses are suitable in the detection of the presence of different subpopulations within a breed [9]. Furthermore, Wright’s F-statistics provides information about the structuring of a population, which, in turn, sheds light on the genetic diversity within a breed [9,12].

In the context of local farm animal breeds, no comprehensive and current overview about the assessment of genetic diversity based on genomic data is available to date. Therefore, the aim of this review is to give an overview about the use of genotyping techniques for assessing genetic diversity in local farm animal breeds worldwide. The following questions were considered: (1) What target breeds were included and is above that the inclusion of other breeds documented? (2) What genotyping techniques were used? (3) How can the development of the techniques used over time be described? (4) What diversity parameters were assessed and what programs were used? (5) Are the genotypes of the target breeds publicly available? (6) Is the measurement of defined phenotypes described? 

## 2. Materials and Methods

The search for studies was conducted using websites of relevant journals (Table S1). The keyword “diversity” was used in combination with “local breeds” as search criteria and the publication years ranged from 2005 to 2020. Further requirements were that local farm animal breeds were investigated, and the assessment of diversity was based on genotyping the target animals. Additionally, only studies conducted on cattle, sheep, goats, chickens and pigs were included. Besides, only information regarding the aforementioned aspects of genetic diversity including within- and between-breed parameters, such as heterozygosity and genetic distances, as well as inbreeding and coancestry, were considered. The search for studies was completed by 1 December 2020. Descriptive statistics were produced with Microsoft Excel and SPSS. 

## 3. Results

### 3.1. General Information with Special Consideration of Investigated Breeds

In total, 133 studies on genetic diversity were found that matched the search criteria: 30% on cattle representing the highest proportion, 20% for sheep and 17% for chickens, pigs and goats, respectively. The investigated breeds had a geographical distribution of 38% (Asia), 36% (Europe), 16% (Africa) and 8% (North and South American), and the remaining 2% included only three studies spread across different countries.

In almost half of the studies (43%), between one and five local target breeds were investigated. In 37% of the studies, six to 15 local breeds were considered, and in 13%, it was more than 15 breeds. Additionally, in 10% of cases, it was not quite clear how many local breeds were included; in some of these cases, several investigated populations were stated. 

There were not only differences in the number of breeds but also the number of genotyped individuals varied across the included studies. Overall, 15–6635 individuals of local breeds were genotyped per study, whereas, in more than half of the studies (61%), the number of genotyped animals ranged between 101 and 500. In 21% of included studies, the number of individuals was 100 or lower, and in 18%, it was above 500. However, these data only encompass the target breeds.

The target breeds were designated differently. Some authors call them “local breeds”; however, a variety of other terms such as “native”, “exotic”, “indigenous”, “heritage” or “endangered” were found. 

### 3.2. Non Target Breeds

From a total of 133 studies, 45 had documented the inclusion of genomic data from breeds other than the target breed. Mostly, this aimed to shed light on the parameters of between-breed diversity. Therefore, genotypes of other local, commercial or wild breeds were commonly found. These genotyping data were usually taken from open databases or previous studies. However, the source of the data is, in some cases, not clearly described. For example, only 15 studies indicated a reference database, e.g., Dryad or GenBank. There were also differences in the origin of the added breeds. Some originated from the same country or the same region while others were from different parts of the world. 

### 3.3. Genotyping Techniques

**Figure 1:**
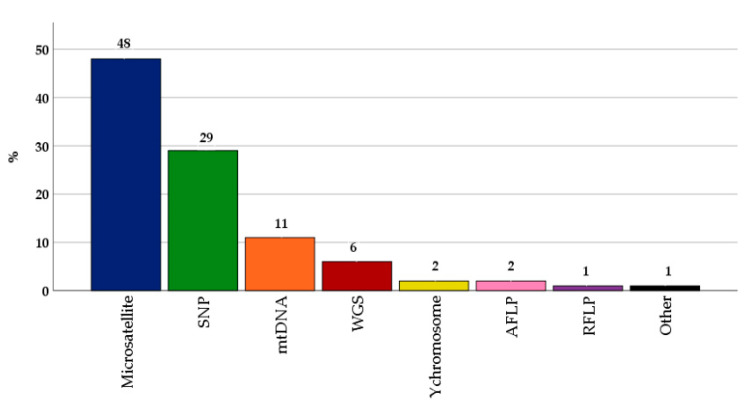
Distribution of the use of genotyping techniques in genetic diversity studies of local farm animal breeds between 2005 and 2020 (n = 140).

Microsatellites were the most (48%) frequently used markers for genotyping local farm animal breeds (Figure 1). In total, the number of utilized microsatellites per study varied from eight to 105. However, in most of the studies, the number of microsatellites used ranged from 15 to 30. Furthermore, 29% of the genotyping was conducted using SNP-arrays. Figure 1 shows that other techniques such as mtDNA or y-chromosome analysis, WGS, AFLP and RFLP had a share of less than 11%. In seven of the 133 analyzed studies, two of the mentioned techniques were combined for genotyping. In most of these cases, it was a combination of mtDNA analysis and the use of microsatellites. Therefore, the number of different genotyping techniques reached 140 (Figure 1).

### 3.4. Changes of the Use of Genotyping Techniques over Time

**Figure 2:**
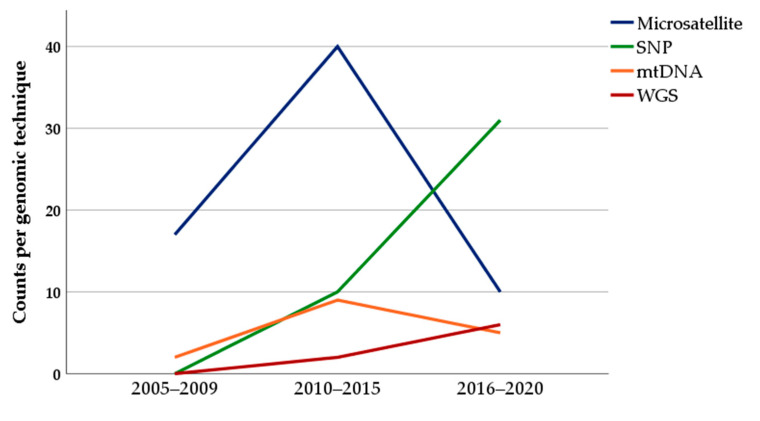
Development of the use of microsatellite, SNP-arrays, mtDNA analysis and whole-genome sequencing (WGS) for genotyping local farm animal breeds between 2005 and 2020 (n = 133 included studies).

Prior to the year 2010, the share of microsatellite markers in terms of genotyping of local farm animals was about 81%, while the contributions of mtDNA, SNP-array and WGS were minimal (Figure 2). Between 2010 and 2015, genotyping based on microsatellites still dominated the field but with a slightly reduced shared percentage of 63%. At the same time, the share of genotyping based on SNP-arrays and on mtDNA analysis rose to 16% and 14%, respectively. As shown in Figure 2, between 2015 and 2020, genotyping based on SNP-arrays dominated with a share of 55%, and the use of microsatellites decreased to an amount of 18%. In addition, the share of mtDNA analyses decreased to about 9%, whereas genotyping based on WGS peaked at 11% for the first time. Other techniques including y-chromosome AFLP and RFLP analysis were found with a range of 1–3% in the period under consideration. 

### 3.5. Diversity Parameters and Evaluation Software

For assessing genetic diversity in local breeds, a wide range of different parameters were found to have been used in the identified studies. These parameters were used to measure diversity within or between breeds, as well as inbreeding and coancestry. As shown in Table 1, Table 2, Table 3 and Table 4, a variety of software programs were used to compute the parameters of interest (for citations, see Table S2).

The analysis of 68 studies dealing with microsatellite data revealed a high frequency (90%) of use of the Wright’s F-statistics as a measure of diversity (Table 1). Observed and expected heterozygosity (H_O_, H_E_) were also measured frequently (85%), whereas other parameters such as effective population size (N_E_) or multidimensional scaling (MDS) were investigated in fewer studies. A total of 32 different software programs were employed in the evaluation of diversity across the various studies.

Table 2 summarizes the findings of 41 studies dealing with genomic data based on SNP-arrays. The investigation of population structure using admixture analysis of SNP data was most frequently (85%) conducted. Similar to the microsatellite analysis, the analysis of SNP-array also featured Wright’s F-statistics as a frequently (78%) measured parameter. Neighbor-joining-tree and different inbreeding coefficients were also computed in more of half the studies, whereas other parameters, such as private alleles or the mean number of alleles, were investigated in just a few studies. Genomic evaluation on the basis of SNP-arrays made use of 24 different software programs, among which Plink [81] (v1.7 and v1.9) was predominant.

A total of 16 studies were found to have been conducted dealing with genomic data based on mtDNA analysis and all these studies shed light on phylogenetic structure (Table 3). Haplotype and nucleotide diversity were also measured very often (81%), whereas parameters such as linkage disequilibrium (LD) and MDS were computed less frequently. Overall, 15 different evaluation programs were used in the identified mtDNA-based studies.

In 100% of studies dealing with genomic data based on WGS (n = 7), principal component analysis (PCA) was conducted (Table 4). Additionally, phylogenetic and population structures as well as LD-based analyses were measured in more than half of the studies. Furthermore, other parameters such as N_E_ and polymorphic information content (PIC) were less frequently investigated. A total of 12 different evaluation programs were involved in the WGS-based evaluations.

In the three studies in which animals were genotyped based on y-chromosome analysis, Wright’s F-statistics and median network were the most frequently computed parameters. Furthermore, haplotype frequency and diversity as well as analysis of molecular variance (AMOVA) and MDS were assessed, and Arlequin [140] was the most used program [54,141,142]. In studies with genomic data based on AFLP analysis, expected heterozygosity, allele frequencies and genetic distances were most often investigated. For this latter genome-based technique, different programs were used, with Popgene [143], Dispan (Ota, 1993) and Phylip [144] playing important roles [145,146,147]. The one study that involved genomic data based on RFLP analysis also assessed different already mentioned diversity parameters, with the application of Popgene and Cervus [148,149]. Giovambattista et al. [150] analyzed genomic data based on BoLA-DRB3 alleles genotyping. With this, different parameters were assessed while using different programs to shed light on the genetic diversity of local cattle populations in Myanmar. 

### 3.6. Availability of Genomic Data and Phenotype

Genomic data from the target local breeds are publicly available for 20 of the studies included. An overview about breeds and sources of genomic data can be found in Table S3. Defined measurements of phenotypic traits were found in only 10 studies [38,48,66,67,77,79,93,97,100,136]. 

## 4. Discussion

The number of local farm animal breeds is declining worldwide, although within- and between-breed diversity is important for future challenges in livestock production systems [1,9]. The present study has reviewed 133 published studies of local farm animal breeds with genetic diversity determined using different genotyping techniques. Six questions were considered to provide a comprehensive overview about target breeds, genotyping techniques and investigated diversity parameters. In the following, the results are discussed in detail and conclusions have been drawn.

### 4.1. Target and Non Target Breeds

The observed frequency of investigated species in diversity studies (cattle > sheep > chickens/pigs/goats) is only partially consistent with the results of the FAO [3] on the worldwide number of reported local breeds. According to the FAO [3] local chicken, followed by sheep and cattle, formed the highest variability of breeds, whereas local pig and goat breeds were only found in small numbers. This slightly different order may be due to the fact that, especially in the case of poultry, the use of just a few commercial hybrids is dominant worldwide [151]. This might be one reason for the low level of research interest in the area of local chicken breeds. According to the FAO [3], most of the local cattle, sheep, goat, chicken and pig breeds originated from European countries, followed by Asian and African countries. Pig breeds are mainly found in Asia, followed by Europe and America [3]. Conversely, the present results show that most of the breeds were originated from Asian, followed by European and African, countries. In summary, this comparison shows that a high number of local breeds being found in a specific region seems not to be automatically connected to a high research interest in conserving them. 

Most of the studies (80%) investigated one to 15 target breeds. Furthermore, the proportion of investigated individuals in the included studies was limited. In most investigations, the number of individuals per study was lower than 500, and, for 21% of investigations, this number was less than 100. Usually, these breeds were kept in smallholder structures. Therefore, the choice of appropriate individuals and sampling was challenging. Furthermore, the inclusion of a larger number of local target breeds as well as individuals was possible only in the case of a few studies. In addition, it is important to note that studies were included from a time when the use of genotyping techniques was more expensive than it is currently [6]. Due to decreasing costs, the number of genotyped individuals in such studies could rise, thus increasing the representativeness of the results in the future. Nevertheless, one third of the studies considered included genotyping data beyond the target breeds. Therefore, for some investigations, such as population structure analysis, the samples are at least slightly larger. 

A crucial point of discussion concerns the designation of breeds. As described, not all target breeds were designated as “local breeds”. According to the FAO [1] the term “local breed” is defined as a breed that is adapted to local conditions and a particular production environment. However, for terms such as “native”, “indigenous” or “heritage”, no corresponding definitions are known. Additionally, these terms do not provide information about the risk-status of the breeds. One exception is if the target breeds are designated as “endangered”. According to the risk-status scheme of the FAO [3], an endangered breed is classified as at risk. Additionally, in some of the studies, the risk-status of the target breeds is described in detail or it is stated that a survey is used to prioritize breeds for conservation [14,27,29,52,65,98,117]. Other authors used the diversity measurements to assess the genetic contribution of single breeds to the total genetic diversity of a set of breeds or populations [29,35,73,74,79]. However, from most studies, it cannot be deduced whether the target breeds are at risk, or to which risk-status they can be assigned.

### 4.2. Genotyping Techniques and Their Changed Use over Time 

The different genotyping techniques that were identified in the present work are largely consistent with the techniques used for diversity studies in livestock described by Yaro et al. [6]. However, in contrast to the results of Yaro et al. [6], no studies were found in which local breeds were genotyped based on RAPD analysis. Nevertheless, the authors have given this technique little importance in context of animal-based molecular genetic studies [6]. The description of the changes in the use of genotyping techniques in diversity studies of local breeds is also mostly consistent with the documented history of marker-assisted genotyping [5,6]. One exception is the recent use of RFLP marker analysis, although Yaro et al. [6] gave less importance to this technique since the introduction of genotyping based on microsatellites. However, it is important to note that there was only one study found that involved the use of RFLP marker analysis and, therefore, the present results may not be representative. Furthermore, a declining importance of genotyping based on y-chromosome and mtDNA analysis is described in context of genotyping farm animal breeds since the introduction of microsatellite and SNP-arrays [6]. In contrast, the present results show that mtDNA and y-chromosome analysis were mostly used in the period between 2010 and 2015, and also in recent years, although to a lesser extent. However, in the years between 2010 and 2015 the highest use of genotyping based on microsatellite markers can be drawn from the present results. Yaro et al. [6] emphasized the importance of using microsatellites for assessing diversity in farm animal breeds, especially in developing countries, where there is often a lack of suitable infrastructure for elaborate analyses. The authors attributed this to the low costs and the reduced effort requirements of this genotyping technique [6]. Furthermore, various advantages of genotyping based on SNP-arrays were described, highlighting the possibility to give deeper insights into the genetic architecture because of the higher marker density [6]. This is reflected by the increased use of SNP-arrays in the included studies after the year 2015, when the amount of genotyping based on microsatellite declined. WGS was less frequently (6%) used in the present studies and only increased slightly after 2015, although the use of WGS for genotyping farm animal breeds has been described since the year 2007 [6]. However, it is necessary to add that the use of WGS was associated with high costs and effort, especially in the beginning [6]. This could be a reason for the infrequent and fairly recent use of the WGS technique in local breeds. The use of WGS can be advantageous in the sense that it prevents ascertainment bias, which is mostly associated with SNP data analysis of target breeds that differ genetically from a reference population [5,6,7]. 

### 4.3. Diversity Parameters and Software Programs

The present results show that a wide range of different parameters were computed for measuring the genetic diversity of local farm animal breeds. Among the frequently used parameters, observed and expected heterozygosity and Wright’s F-statistics have been widely considered suitable to assess genetic diversity within a breed and to shed light on genetic structure of subdivided populations [5,9,11,12]. The assessment of genetic differentiation between breeds was mostly conducted based on genetic distances and visualized using the neighbour-joining method, as described by Toro et al. [9] as well as Marsjan and Oldenbroek [11]. Additionally, different inbreeding coefficients (F_IS_, F_ROH_) or parameters for the assessment of coancestry (e.g., kinship coefficients) were computed. This is consistent with the findings of Eding and Meuwissen [8] and Toro et al. [9], which emphasize that the assessment of marker-estimated kinships can provide information on genetic diversity within and between breeds, especially in the context of conservation. It is also evident from the present results that the frequency of assessed diversity parameters depends on the genotyping technique. For example, the assessment of H_O_ and H_E_ was calculated less frequently in studies where animals were genotyped based with SNP-arrays than in case of the use of microsatellite. With the introduction of high-density SNP-arrays, there is probably less importance attached to a single marker in the context of linkage. Due to high density requirements, inbreeding coefficients based on runs of homozygosity (F_ROH_) were only computed when using SNP-arrays. The reasoning behind this may be that research around the analysis of runs of homozygosity in the context of farm animal breeds coincided with a period of growing use of high-density SNP-arrays [152].

The development of new genotyping techniques is accompanied by an increase in the number of software programs for computing parameters of genetic diversity. The presented variety of these programs is almost consistent with the results of Boettcher et al. [4], who have compiled a list of suitable programs for prioritizing breeds. The authors also pointed out that some programs, e.g., Arlequin [140] and Popgene [143], are suitable for the calculation of several diversity parameters. However, in most studies, several programs have to be used per study because not all applications can be covered by one program [4]. Most of the results of the reviewed studies were published shortly before the Plink software program was used extensively [152]. Plink was developed to facilitate the handling of large datasets in human whole-genome association studies and published in 2007 [81]. Later, the program provided the platform for other was uses including the assessment of runs of homozygosity as a measure of autozygosity [152]. Peripolli et al. [152] viewed Plink as superior to other programs such as GERMLINE [153] and BEAGLE [154], especially with regard to the analysis of runs of homozygosity. In fact, Plink is suitable for calculating most of the parameters described and it is replacing the use of a wide variety of other programs [155]. It is, therefore, not surprising that the present study found Plink to be a major program for SNP-based evaluation.

### 4.4. Availability of Genomic Data and Phenotype

At 17%, the availability of the data of target breeds seems to be low. Nevertheless, Groeneveld et al. [156] pointed out the abundance of different databases in the context of genotyping data of farm animal breeds, which, with few exceptions, are not continuously updated. In addition, data compatibility is not always assured and, therefore, the integration of genotypes from databases into an ongoing study might be challenging [156]. These circumstances may explain why some available data are not used to increase sample sizes and thus benefit from these existing results. Furthermore, only 8% of included studies had defined phenotypes available. This could also be related to the observation that local breeds are usually kept in small numbers in a larger region and, therefore, phenotype recording requires a considerable effort. 

## 5. Conclusions

To be able to handle future challenges in agriculture, it is important to conserve within- and between-breed diversity. The development and use of genotyping techniques enable investigators to obtain deep insights into the parameters of genetic diversity, on which basis breed prioritization, conservation or management decisions are made. The present work provides an overview of the use of genotyping techniques for the assessment of genetic diversity of local farm animal breeds. Since 2005, a significant number of different (mostly cattle) breeds from all over the world were part of diversity studies. For this, different genotyping techniques were used to calculate various diversity parameters. At 48%, microsatellite techniques were dominant, although the use of SNP-arrays and WGS increased between 2016 and 2020. Changes in genotyping techniques used in the different studies over time may reflect advances in the technologies that underlie these techniques, but also successes made in the development of computational algorithms and software programs. The widespread use of microsatellites in the field of local breeds is possibly due to their low costs and low required effort. Nevertheless, high density SNP-arrays and WGS provide various advantages in assessing diversity of local breeds, such as the avoidance of ascertainment bias and a significantly higher information content, with ever decreasing costs at the same time. Therefore, preference should be given to these techniques whenever possible. The results show that, in many cases, small sample sizes of individuals were genotyped. Therefore, the documentation of publicly available genotypes and recorded phenotypes provides the possibility to enlarge datasets and, therefore, improve research in this field in the future. In addition, larger sample sizes can also be achieved by imputing data sets from expensive or more complex genotyping of a few individuals. Assessing genetic diversity of breeds as accurately as possible is important for conservation purposes. The extent of diversity of one breed or a set of populations is the basis for their prioritization and helps in the development of targeted conservation strategies. In summary, the present review provides an overview of the development in the field of diversity studies and offers a crucial fundament for the application of genotyping techniques in the future. This can contribute to an improvement in local farm animal breed conservation. 

## Figures and Tables

**Table 1:** Percentage of computed diversity parameters in 68 studies * involving microsatellite analysis, and the respective software programs used (for citations, see Table S2).

Parameter	n	%	Software
Wright’s F-statistics	61	90	Arlequin, Cervus, FSTAT, GDA, GenAlEx, Genepop, Genetix, HP-Rare, MolKin, POPGENE, Populations, SAS
Observed Heterozygosity	58	85	Arlequin, Cervus, GenAlEx, Genetix, FSTAT, Microsatellite Toolkit, MolKin, PHYLIP, POPGENE
Expected Heterozygosity	58	85	Arlequin, Cervus, FSTAT, GenAlEx, Genetix, Microsatellite Toolkit, MolKin, POPGENE, PHYLIP
Population structure/Admixture	51	75	BAPS, CLUMPP, Distruct, Genetix, Leadmix, Structure
Genetic distances	49	72	Arlequin, Dispan, Genetix, MolKin, Phase, PHYLIP, POPGENE, Populations
Effective/mean number of alleles	48	71	Arlequin, FSTAT, GenAlEx, Genetix, Microsatellite Toolkit, MolKin, POPGENE
Hardy–Weinberg equilibrium test	48	71	Arlequin, Cervus, GenAlEx, Genepop, POPGENE, SAS
Neighbor-joining-/phylogenetic tree	37	54	Dispan, Mega, PHYLIP, r, SplitsTree
Allele frequencies	36	53	Cervus, FSTAT, GenAlEx, Genetix, Genepop, Microsatellite Toolkit, MolKin, Populations
Allelic richness	28	41	FSTAT, GenAlEx, HP-RARE, POPGENE
Polymorphic information content	23	34	Cervus, Excel, Microsatellite Toolkit, MolKin
Analysis of molecular variance	16	24	Arlequin, GenAlEx
Principal component analysis	15	22	Fortran, GenAlEx, MVSP, r, SAS, SPSS, XLSTAT
Private alleles	12	18	FSTAT, GenAlEx, GDA, HP-RARE, Microsatellite Toolkit, Populations
Linkage disequilibrium	10	15	Genepop, SAS
Null alleles	8	12	Cervus, FreeNA, Micro-Checker
Genetic relationships/coancestry	8	12	Admixture, Genetix, MolKin, r
Gene diversity	5	7	FSTAT, Genetix, Microsatellite Toolkit
Proportion of shared alleles	5	7	Microstat
Effective population size	4	6	Cervus, GenAlEx, POPGENE
Multidimensional scaling	4	6	r, DARwin, GenAlEx
Allelic diversity per locus	3	4	Microsatellite Toolkit, MolKin
Multiple co-inertia analysis	2	3	r
Percentage of polymorphic loci	1	1	POPGENE
* References	[13,14,15,16,17,18,19,20,21,22,23,24,25,26,27,28,29,30,31,32,33,34,35,36,37,38,39,40,41,42,43,44,45,46,47,48,49,50,51,52,53,54,55,56,57,58,59,60,61,62,63,64,65,66,67,68,69,70,71,72,73,74,75,76,77,78,79,80]		

**Table 2:** Percentage of computed diversity parameters in 41 studies * based on SNP-arrays and respective software programs used (for citations, see Table S2).

Parameter	n	%	Software
Population structure/Admixture	35	85	Admixture, fastSTRUCTURE, Python, Structure, TreeMix
Wright’s F-statistics	32	78	Arlequin, Genepop, Golden Helix SNP Variation Suite, Power marker, Plink, r, VCFtools
Neighbor net/ neighbor-joining-/max. likelihood tree	28	68	Arlequin, hapFLK, Mega, PHYLIP, r, RAxML, SplitsTree, TreeMix
F_ROH_/other inbreeding coefficients than F_IS_	28	68	Arlequin, Haploview, Plink, r
Principal component analysis	26	63	Eigensoft, Eigenstrat, GCTA, Golden Helix SNP variation Suite, Plink, r
Linkage disequilibrium	26	63	Haploview, Plink, r, SNeP
Expected heterozygosity	26	63	Arlequin, Plink, r
Observed heterozygosity	23	56	Arlequin, Plink, r
Effective population size	21	51	NeESTIMATOR, Plink, r, SNeP
Genetic distances	20	49	Arlequin, hapFLK, Genepop, PHYLIP, Plink, Power marker, r
Multidimensional scaling	15	37	Haploview, Plink, r,
relationship/ coancestry	11	27	Admixture, GCTA, Haploview, Plink, r,
Allelic richness	10	24	Adze, r
Analysis of molecular variance	7	17	Arlequin
Proportion of polymorphic markers/loci	6	15	Plink, r
Allele frequency	5	12	Plink, Golden Helix SNP variation Suite
Hardy–Weinberg equilibrium test	4	10	Plink
Proportion of shared alleles	3	7	Plink
Private alleles	1	2	
Total/mean number of alleles	1	2	
* References	[82,83,84,85,86,87,88,89,90,91,92,93,94,95,96,97,98,99,100,101,102,103,104,105,106,107,108,109,110,111,112,113,114,115,116,117,118,119,120,121,122]		

**Table 3:** Percentage of computed diversity parameters in 16 studies * based on mtDNA analysis and respective software programs used (for citations, see Table S2).

Parameter	n	%	Software
Neighbor-joining-/phylogenetic/max. likelihood tree	16	100	Cipres, MEGA, MrBAYES, Network, Populations, SplitsTree
Haplotype diversity	13	81	Arlequin, DnaSP, SPSS, Vcftools
Nucleotide diversity	13	81	Arlequin, DnaSP, SPSS
Genetic distances	6	38	Arlequin, DnaSP, MEGA, Populations
Wrights or Fu’s F-statistics	5	31	Arlequin, Plink, r
Analysis of molecular variance	4	25	Arlequin, SAMOVA
Number of polymorphic sites	3	19	DnaSP
Principal component analysis	3	19	Plink, r, SAS, SPSS
Observed heterozygosity	2	13	Vcftools
Allele frequencies	2	13	Genetix, Vcftools
Relatedness	2	13	MEGA, Vcftools
Genetic diversity	2	13	DnaSP
Expected heterozygosity	1	6	Genetix
Linkage disequilibrium	1	6	Vcftools
Multidimensional scaling	1	6	XLSTAT
Multiple co-inertia analysis	1	6	r
* References	[25,32,40,67,69,75,123,124,125,126,127,128,129,130,131,132]		

**Table 4:** Percentage of computed diversity parameters in seven studies * based on whole-genome sequencing and respective software programs used (for citations, see Table S2).

Parameter	n	%	Software
Principal component analysis	7	100	Eigensoft, GCTA, Plink
Neighbor-joining-/phylogenetic tree	5	71	MEGA, PHYLIP
Linkage disequilibrium	5	71	Plink, Vcftools
Population structure	4	57	Admixture, Structure
Expected heterozygosity	3	43	Arlequin, Plink
Observed heterozygosity	3	43	Arlequin, Plink
Genetic distances	3	43	FSTAT, MEGA, Plink
Wright’s F-statistics	3	43	Arlequin, BioPerl, FSTAT, Vcftools
Allelic richness	3	38	Adze
Nucleotide diversity	2	29	BioPerl, Vcftools
Proportion of polymorphic sites/marker	2	29	BioPerl, Plink
Effective population size	2	29	Plink
Allele frequencies	1	14	FSTAT
F_ROH_/Inbreeding coefficients other than F_IS_	1	14	Plink
* References	[133,134,135,136,137,138,139]		

## Supplementary Materials

The following are available online at https://www.mdpi.com/article/10.3390/ani11072016/s1, Table S1: Journals searched for suitable studies investigating genetic diversity based on genotyping of local farm animal breeds using the keywords “diversity” and “local breed” by 1 December 2020; Table S2: Citations of mentioned software programs suitable for assessing genetic diversity parameters; Table S3: Publicly available raw data of different local cattle, sheep, goat, chicken and pig breeds based on different genotyping techniques.
