# Supplementary material for: An Overview of the Use of Genotyping Techniques for Assessing Genetic Diversity in Local Farm Animal Breeds
Source: Animals (Basel). 2021 Jul 6;11(7):2016. doi: 10.3390/ani11072016 (PMC8300386; doi:10.3390/ani11072016)
Supplement: Supplementary file 1 [file animals-11-02016-s001.zip › Table_S1.pdf]

animals

MDPI

Table S1: Journals searched for suitable studies investigating genetic diversity based on genotyping of local farm animal breeds using the keywords "diversity" and "local breed" by Dec. 1, 2020.

| |
|---|
| Livestock Science |
| Journal of Animal Science |
| Animal Production |
| BMC Genetics |
| Frontiers in Genetics |
| Global Ecology and Conservation |
| Reproduction in Domestic Animals |
| Diversity |
| Genetics Selection Evolution |
| Animal Genetic Resources |
| Animal Genetics |
| Animal Science Journal |
| Genetics |
| Genome Research |
| Genetics Research |
| Heredity |
| Journal of Animal Breeding and Genetics |
| Global Journal of Animal Breeding and Genetics |
| International Journal of Animal Breeding and Genetics |
| Journal of Dairy Science |
| Genetic Resources |
| PlosOne |
| Conservation Biology |
