# Supplementary material for: An Overview of the Use of Genotyping Techniques for Assessing Genetic Diversity in Local Farm Animal Breeds
Source: Animals (Basel). 2021 Jul 6;11(7):2016. doi: 10.3390/ani11072016 (PMC8300386; doi:10.3390/ani11072016)
Supplement: Supplementary file 1 [file animals-11-02016-s001.zip › Table_S2.pdf]

animals

MDPI

Table S2: Citations of mentioned software programs suitable for assessing genetic diversity parameters

Program	Authors/Citation
Admixture	Alexander, D.H.; Novembre, J.; Lange, K. Fast model-based estimation of ancestry in unrelated individuals. *Genome Research* **2009**, 19, 1655-1664, doi:10.1101/gr.094052.109.
Adze	Szpiech, Z. A.; Jakobsson, M.; Rosenberg, N. A. ADZE: a rarefaction approach for counting alleles private to combinations of populations. *Bioinformatics* **2008**, 24, 2498-2504, doi:10.1093/bioinformatics/btn478
Arlequin	Excoffier, L.; Lischer, H.E. L. Arlequin suite ver 3.5: A new series of programs to perform population genetics analyses under Linux and Windows. *Molecular Ecology Resources* **2010**, 10, 564-567, doi:10.1111/j.1755-0998.2010.02847.x.
BAPS	Corander, J.; Marttinen, P. Bayesian identification of admixture events using multi-locus molecular markers. *Molecular Ecology* **2006**, 15, 2833-2843, doi:10.1111/j.1365-294X.2006.02994.x (Please note: For the calculation of other parameters different citations must be given.)
BioPerl	Stajich, J. E.; Block, D.; Boulez, K.; Brenner, S. E.; Chervitz, S. A.; Dagdigian, C.; Fuellen, G.; Gilbert, J. G.; Korf, I.; Lapp, H.; Lehväslaiho, H.; Matsalla, C.; Mungall, C. J.; Osborne, B. I.; Pocock, M. R.; Schattner, P.; Senger, M.; Stein, L. D.; Stupka, E.; Wilkinson, M. D.; Birney, E. The Bioperl toolkit: Perl modules for the life sciences. *Genome Research* **2002**, 12, 1611-8, doi: 10.1101/gr.361602
Cervus	Kalinowski, S.T.; Taper, M.L.; Marshall, T.C. Revising how the computer program CERVUS accommodates genotyping error increases success in paternity assignment. *Molecular Ecology* **2007**, 16, 1099-1106, doi: 10.1111/j.1365-294X.2007.03089.x.
Cipres	Miller, M. A.; Pfeiffer, W.; Schwartz, T. Creating the CIPRES Science Gateway for inference of large phylogenetic trees. Proceedings of the Gateway Computing Environments Workshop (GCE), 14 Nov. 2010, New Orleans, LA, 1- 8.
CLUMPP	Jakobsson, M.; Rosenberg, N.A. CLUMPP: a cluster matching and permutation program for dealing with label switching and multimodality in analysis of population structure. *Bioinformatics* **2007**, 23, 1801-1806, doi: 10.1093/bioinformatics/btm233.
DARwin	Perrier, X.; Jacquemoud-Collet, J.P. (2006) DARwin software http://darwin.cirad.fr/
DISPAN	Copyright 1993 by Tatsuya Ota and the Pennsylvania State University
Distruct	Rosenberg, N.A. DISTRUCT: a program for the graphical display of population structure. *Molecular Ecology Notes*, **2004**, 4, 137-138, doi: 10.1046/j.1471-8286.2003.00566.x.
DnaSP	Rozas, J.; Ferrer-Mata, A.; Sánchez-DelBarrio, J. C.; Guirao-Rico, S.; Librado, P.; Ramos-Onsins, S. E.; Sánchez-Gracia, A. DnaSP 6: DNA Sequence Polymorphism Analysis of Large Datasets. *Molecular Biology and Evolution* **2017**, 34, 3299-3302, doi:10.1093/molbev/msx248
Eigensoft/Eigenstrat	Price, A.; Patterson, N. J.; Plenge, R. M.; Weinblatt, M. E.; Shadick, N. A.; Reich, D. Principal components analysis corrects for stratification in genome-wide association studies. *Nature Genetics* **2006**, 38, 904-909, doi:10.1038/ng1847

	Patterson, N.; Price, A.; Reich, D. Population Structure and Eigenanalysis. *Plos Genetics* **2006**, 2, doi:10.1371/journal.pgen.0020190
fastSTRUCTURE	Raj, A.; Stephens, M.; Pritchard, J. K. fastSTRUCTURE: Variational Inference of Population Structure in Large SNP Data Sets. *Genetics* **2014**, 197, 573-589, doi:10.1534/genetics.114.164350
FreeNA	Chapuis, M.-P.; Estoup, A. Microsatellite null alleles and estimation of population differentiation. *Molecular Biology and Evolution* **2007**, 24, 621-631, doi:10.1093/molbev/msl191.
FSTAT	Goudet, J. FSTAT (Version 1.2): A Computer Program to Calculate F-Statistics. *Journal of Heredity* **1995**, 86, 485-486, doi:10.1093/oxfordjournals.jhered.a111627.
GCTA	Yang, J.; Lee, S. H.; Goddard, M. E.; Visscher, P. M. GCTA: a tool for genome-wide complex trait analysis. *American Journal of Human Genetics* **2011**, 88, 76-82, doi:10.1016/j.ajhg.2010.11.011
GDA	Designed by Paul O. Lewis and Dmitri Zaykin to accompany the book: Weir, B. S. 1996. Genetic Data Analysis. 2nd ed. Sinauer Associates, Sunderland, Massachusetts. 376 pages.
GenAlEx	Peakall, R.; Smouse, P.E. GENALEX 6: genetic analysis in Excel. Population genetic software for teaching and research. *Molecular Ecology Notes* **2006**, 6, 288-295, doi:10.1111/j.1471-8286.2005.01155.x.
	Peakall, R.; Smouse, P.E. GenAlEx 6.5: genetic analysis in Excel. Population genetic software for teaching and research-an update. *Bioinformatics* **2012**, 28, 2537-2539, doi: 10.1093/bioinformatics/bts460.
Genepop	Raymond, M.; Rousset, F. GENEPOP (version 1.2): population genetics software for exact tests and ecumenicism. *Journal of Heredity*, **1995**, 86, 248-249, doi:10.1093/oxfordjournals.jhered.a111573.
	Rousset, F. Genepop'007: a complete reimplementation of the Genepop software for Windows and Linux. *Mol. Ecol. Resources* **2008**, 8, 103-106, doi:10.1111/j.1471-8286.2007.01931.x.
Genetix	Belkhir K., Borsa P., Chikhi L., Raufaste N. & Bonhomme F. 1996-2004 GENETIX 4.05, logiciel sous Windows TM pour la génétique des populations. Laboratoire Génome, Populations, Interactions, CNRS UMR 5000, Université de Montpellier II, Montpellier (France)
Golden Helix SNP Variation Suite	Bozeman, MT: Golden Helix, Inc. Available from http://www.goldenhelix.com
hapFLK	Fariello, M. I.; Boitard, S.; Naya, H.; SanCristobal, M.; Servin, B. Detecting Signatures of Selection Through Haplotype Differentiation Among Hierarchically Structured Populations. *Genetics* **2013**, 193, 929-941, doi: 10.1534/genetics.112.147231
Haploview	Barrett, J. C.; Fry, B.; Maller, J.; Daly, M. J. Haploview: analysis and visualization of LD and haplotype maps. *Bioinformatics* **2005,** 21, 263-5, doi:10.1093/bioinformatics/bth457
HP-Rare	Kalinowski, S.T. HP-Rare 1.0 : a computer program for performing rarefaction on measures of allelic richness. *Molecular Ecology Notes* **2005**, 5, 187-189, doi:10.1111/j.1471-8286.2004.00845.x
Leadmix	Wang, J. Maximum Likelihood Estimation of Admixture Proportions from Genetic Data. *Genetics* **2003**, 164, 747-765
Mega	Tamura, K.; Stecher, G.; Kumar, S. MEGA11: Molecular Evolutionary Genetics Analysis version 11. *Molecular Biology and Evolution* **2021**, 23, doi: 10.1093/molbev/msab120 (Please note: For the indication of other versions another citation must be given).

Micro-Checker	Van Oosterhout, C.; Hutchinson, W.F.; Wills, D.P.M.; Shipley, P. micro-checker: software for identifying and correcting genotyping errors in microsatellite data. *Molecular Ecology Notes* **2004**, 4, 535-538, doi: 10.1111/j.1471-8286.2004.00684.x.
Microsatellite Toolkit	No actual citation available.
MolKin	Gutiérrez, J.P.; Royo, L.J.; Álvarez, I.; Goyache, F. MolKin v2.0: a computer program for genetic analysis of populations using molecular coancestry information. *Journal of Heredity* **2005**, 96, 718-721, doi:10.1093/jhered/esi118
MrBAYES	Huelsenbeck, J. P.; Ronquist, F. MRBAYES: Bayesian inference of phylogeny. *Bioinformatics* **2001**, 17, 754-755, doi:10.1093/bioinformatics/17.8.754<br>Ronquist, F.; Huelsenbeck, J. P. MRBAYES 3: Bayesian phylogenetic inference under mixed models. *Bioinformatics* **2003**, 19, 1572-1574 doi: 10.1093/bioinformatics/btg180
MVSP	Kovach, W.L., 2007. MVSP - A MultiVariate Statistical Package for Windows, ver. 3.1. Kovach Computing Services, Pentraeth, Wales, U.K.
NeESTIMATOR	Do, C.; Waples, R. S.; Peel, D.; Macbeth, G. M.; Tillett, B. J.; Ovenden, J. R. NeEstimator V2: re-implementation of software for the estimation of contemporary effective population size (Ne) from genetic data. *Molecular Ecology Resources* **2014**, 14, 209-214, doi:10.1111/1755-0998.12157
Network	Bandelt, H. J.; Forster, P.; Röhl, A. Median-joining networks for inferring intraspecific phylogenies. *Molecular Biology and Evolution* **1999**, 16, 37-48, doi:10.1093/oxfordjournals.molbev.a026036
Plink	Purcell, S.; Neale, B.; Todd-Brown, K.; Thomas, L.; Ferreira, M. A. R.; Bender, D.; Maller, J.; Sklar, P.; Bakker, P. I. W. de; Daly, M. J.; et al. PLINK: a tool set for whole-genome association and population-based linkage analyses. *American Journal of Human Genetics* ***2007***, 81, 559-575, doi:10.1086/519795
Phase	Stephens, M.; Smith, N. J., Donnelly, P. A new statistical method for haplotype reconstruction from population data. *American Journal of Human Genetics* **2001**, 68, 978-989<br>Stephens, M.; Scheet, P. Accounting for decay of linkage disequilibrium in haplotype inference and missing data imputation. *American Journal of Human Genetics* **2005**, 76, 449-462, doi:10.1086/428594 (Please note: For the calculation of other parameters different citations must be given.)
PHYLIP	Felsenstein, J. PHYLIP - Phylogeny Inference Package (Version 3.2). *Cladistics* **1989**, 5, 164-166
POPGENE	Yeh, F.C.; Boyle, T.J.B. Population genetic analysis of co-dominant and dominant markers and quantitative traits. *Belgian Journal of Botany* **1997**, 129, 157
Populations	Populations, 1.2.30 Copyright (C) 1999, Olivier Langella, CNRS UPR9034
PowerMarker	Homepage: https://brcwebportal.cos.ncsu.edu/powermarker/index.html Citation information can be obtained by selecting Help|How To Cite PowerMarker in the PowerMarker program.
RAxML	Stamatakis, A. RAxML Version 8: A tool for Phylogenetic Analysis and Post-Analysis of Large Phylogenies. *Bioinformatics* **2014**, 30, 1312-3, doi: 10.1093/bioinformatics/btu033
SAMOVA	Dupanloup, I.; Schneider, S.; Excoffier, L. A simulated annealing approach to define the genetic structure of populations. *Molecular Ecology* **2002**, 11, 2571-81, doi:10.1046/j.1365-294x.2002.01650.x
SNeP	Barbato, M.; Orozco-terWengel, P.; Tapio, M.; Bruford, M. W.

	SNeP: a tool to estimate trends in recent effective population size trajectories using genome-wide SNP data. *Frontiers in Genetics* **2015**, 6, 109, doi:10.3389/fgene.2015.00109
SplitsTree	Huson, D.H.; Bryant, D. Application of Phylogenetic Networks in Evolutionary Studies. *Molecular Biology and Evolution* **2006**, 23, 254-267, doi:10.1093/molbev/msj030
Structure	Pritchard, J.K.; Stephens, M.; Donnelly, P. Inference of Population Structure Using Multilocus Genotype Data. Genetics Society of America 2000, 155, 945-959
TreeMix	Pickrell, J. K.; Pritchard, J. K. Inference of Population Splits and Mixtures from Genome-Wide Allele Frequency Data. *Plos Genetics* **2012**, 8, doi: 10.1371/journal.pgen.1002967
VCFtools	Danecek, P.; Auton, A.; Abecasis, G.; Albers, C. A.; Banks, E.; DePristo, M. A.; Handsaker, R. E.; Lunter, G.; Marth, G. T.; Sherry, S. T.; McVean, G.; Durbin, R.; 1000 Genomes Project Analysis Group. The variant call format and VCFtools. Bioinformatics 2011, 27, 2156-8, doi:10.1093/bioinformatics/btr330
