# Supplementary material for: An Overview of the Use of Genotyping Techniques for Assessing Genetic Diversity in Local Farm Animal Breeds
Source: Animals (Basel). 2021 Jul 6;11(7):2016. doi: 10.3390/ani11072016 (PMC8300386; doi:10.3390/ani11072016)
Supplement: Supplementary file 1 [file animals-11-02016-s001.zip › Table_S3.pdf]

animals

MDPI

Table S3: Publicly available raw data of different local cattle, sheep, chicken, pig and goat breeds based on different genotyping techniques

Breed/Population	Type of Data	Source	Reference
Black Slavonian and Turopolje pig	Illumina PorcineSNP60	Dryad, https://doi.org/10.5061/dryad.wpzgmsbhq	Lukić, B.; Ferenčaković, M.; Šalamon, D.; Čačić, M.; Orehovački, V.; Iacolina, L.; Curik, I.; Cubric-Curik, V. Conservation Genomic Analysis of the Croatian Indigenous Black Slavonian and Turopolje Pig Breeds. *Frontiers in Genetics* **2020**, 11, 1212, doi: 10.3389/fgene.2020.00261
Four Chinese native pig populations	WGS[1]	SRA database, accession no. SRP149063	Qiao, R.; Li, X.; Han, X.; Wang, K.; Lv, G.; Ren, G. Population structure and genetic diversity of four Henan pig populations. *Animal genetics* **2019**, 50, 262–265, doi: 10.1111/age.12775
Göttinger Minipig	WGS	ENA[2], project accession PRJEB36673	Reimer, C.; Ha, N.-T.; Sharifi, A. R.; Geibel, J.; Mikkelsen, L. F.; Schlather, M.; Weigend, S.; Simianer, H. Assessing breed integrity of Göttingen Minipigs. *BMC genomics* **2020**, 21, 308, doi: 10.1186/s12864-020-6590-4
Six indigenous Chinese pig breeds	SNP-data	http://klab.sjtu.edu.cn/iBLUP/genotype_taihu_pigs.zip	Wang, Z.; Chen, Q.; Yang, Y.; Liao, R.; Zhao, J.; Zhang, Z.; Chen, Z.; Zhang, X.; Xue, M.; Yang, H.; Zheng, Y.; Wang, Q.; Pan, Y. Genetic diversity and population structure of six Chinese indigenous pig breeds in the Taihu Lake region revealed by sequencing data. *Animal genetics* **2015**, 46, 697–701, doi: 10.1111/age.12349
One native Chinese pig breed	WGS	NCBI BioProject database, accession PRJNA639223	Wu, F.; Sun, H.; Lu, S.; Gou, X.; Yan, D.; Xu, Z.; Zhang, Z.; Qadri, Q. R.; Zhang, Z.; Wang, Z.; Chen, Q.; Li, M.; Wang, X.; Dong, X.; Wang, Q.; Pan, Y. Genetic Diversity and Selection Signatures Within Diannan Small-Ear Pigs Revealed by Next-Generation Sequencing. *Frontiers in genetics* **2020**, 11, 733, doi: 10.3389/fgene.2020.00733
Three native Korean cattle breeds	Illumina BovineSNP50 BeadChip	Dryad, http://dx.doi.org/10.5061/dryad.55f3s.	Sharma, A.; Lee, S.-H.; Lim, D.; Chai, H.-H.; Choi, B.-H.; Cho, Y. A genome-wide assessment of genetic diversity and population structure of Korean native cattle breeds. *BMC genetics* **2016**, 17, 139, doi: 10.1186/s12863-016-0444-8
Nine Swedish cattle breeds	GeneSeek Genomic Profiler High-Density Bovine 150 K	Dryad, https://datadryad.org/stash /datas et/ doi:10.5061/dryad .wdbrv 15j4	Upadhyay, M.; Eriksson, S.; Mikko, S.; Strandberg, E.; Stålhammar, H.; Groenen, M. A. M.; Crooijmans, R. P. M. A.; Andersson, G.; Johansson, A. M. Genomic relatedness and diversity of Swedish native cattle breeds. *Genetics, selection, evolution* **2019**, 51, 56, doi: 10.1186/s12711-019-0496-0
Two indigenous Irish cattle breeds	Illumina Bovine SNP50 BeadChip	Dryad, https://doi.org/10.5061/dryad.8fk81.	Browett, S.; McHugo, G.; Richardson, I. W.; Magee, D. A.; Park, S. D. E.; Fahey, A. G.; Kearney, J. F.; Correia, C. N.; Randhawa, I. A. S.; MacHugh, D. E. Genomic Characterisation of the Indigenous Irish Kerry Cattle Breed. *Frontiers in genetics* **2018**, 9, 51, doi:10.3389/fgene.2018.00051

Two Finnish and one Siberian cattle breed	WGS	ENA, accession number PRJEB28185	Weldenegodguad, M.; Popov, R.; Pokharel, K.; Ammosov, I.; Ming, Y.; Ivanova, Z.; Kantanen, J. Whole-Genome Sequencing of Three Native Cattle Breeds Originating From the Northernmost Cattle Farming Regions. *Frontiers in genetics* **2018**, 9, 728, doi:10.3389/fgene.2018.00728
Two native cattle breeds from Myanmar	BoLA-DRB3 genotyping	ENA, accession numbers LC466585, LC466586, LC466588	Giovambattista, G.; Moe, K. K.; Polat, M.; Borjigin, L.; Hein, S. T.; Moe, H. H.; Takeshima, S.-N.; Aida, Y. Characterization of bovine MHC DRB3 diversity in global cattle breeds, with a focus on cattle in Myanmar. BMC genetics 2020, 21, 95, doi: 10.1186/s12863-020-00905-8
Five native Swedish sheep breeds	Ovine Infinium HD SNP BeadChip	Dryad, https://datadryad.org/stash/dataset/doi:10.5061/dryad.34	Rochus, C. M.; Jonas, E.; Johansson, A. M. Population structure of five native sheep breeds of Sweden estimated with high density SNP genotypes. *BMC genetics* **2020**, 21, 27, doi:10.1186/s12863-020-0827-8
Five local sheep breeds from Kyrgyzstan	Illumina OvineSNP50 BeadChip	Dryad, https://doi:10.5061/dryad.37pvmcvff	Deniskova, T.; Dotsev, A.; Lushihina, E.; Shakhin, A.; Kunz, E.; Medugorac, I.; Reyer, H.; Wimmers, K.; Khayatzadeh, N.; Sölkner, J.; Sermyagin, A.; Zhunushev, A.; Brem, G.; Zinovieva, N. Population Structure and Genetic Diversity of Sheep Breeds in the Kyrgyzstan. *Frontiers in genetics* **2019**, 10, 1311, doi:10.3389/fgene.2019.01311
Three Belgian sheep breeds	Illumina OvineSNP50 beadchip	Figshare, doi: 10.6084/m9.figshare.11322842	Meyermans, R.; Gorssen, W.; Wijnrocx, K.; Lenstra, J. A.; Vellema, P.; Buys, N.; Janssens, S. Unraveling the genetic diversity of Belgian Milk Sheep using medium-density SNP genotypes. *Animal genetics* **2020**, 51, 258–265, doi: 10.1111/age.12891
Five Ethiopian sheep populations	Illumina Ovine Infinium HD BeadChip	Animal Genome Databases, https://www.animalgenome.org/repository/pub/KORE2017.1122/	Edea, Z.; Dessie, T.; Dadi, H.; Do, K.-T.; Kim, K.-S. Genetic Diversity and Population Structure of Ethiopian Sheep Populations Revealed by High-Density SNP Markers. *Frontiers in genetics* **2017**, 8, 218, doi: 10.3389/fgene.2017.00218
Nine indigenous Arabian sheep breeds	Illumina OvineSNP50 BeadChip	http://widde.toulouse.inra.fr/widde/	Eydivandi, S.; Sahana, G.; Momen, M.; Moradi, M. H.; Schönherz, A. A. Genetic diversity in Iranian indigenous sheep vis-à-vis selected exogenous sheep breeds and wild mouflon. *Animal genetics* **2020**, 51, 772–787, doi:10.1111/age.12985
144 worldwide distributed goat populations	Illumina GoatSNP50 BeadChip	Dryad, https ://doi.org/10.5061/dryad .v8g21 pt	Colli, L.; Milanesi, M.; Talenti, A.; Bertolini, F.; Chen, M.; Crisà, A.; Daly, K. G.; Del Corvo, M.; Guldbrandtsen, B.; Lenstra, J. A.; Rosen, B. D.; Vajana, E.; Catillo, G.; Joost, S.; Nicolazzi, E. L.; Rochat, E.; Rothschild, M. F.; Servin, B.; Sonstegard, T. S.; Steri, R.; van Tassell, C. P.; Ajmone-Marsan, P.; Crepaldi, P.; Stella, A. Genome-wide SNP profiling of worldwide goat populations reveals strong partitioning of diversity and highlights post-domestication migration routes. *Genetics Selection Evolution* **2018**, 50, 58, doi: 10.1186/s12711-018-0422-x

Seven local Spanish goat breeds	Illumina Goat SNP50 BeadChip	Deposited in the Goat AdaptMap database	Manunza, A.; Noce, A.; Serradilla, J. M.; Goyache, F.; Martínez, A.; Capote, J.; Delgado, J. V.; Jordana, J.; Muñoz, E.; Molina, A.; Landi, V.; Pons, A.; Balteanu, V.; Traoré, A.; Vidilla, M.; Sánchez-Rodríguez, M.; Sànchez, A.; Cardoso, T. F.; Amills, M. A genome-wide perspective about the diversity and demographic history of seven Spanish goat breeds. *Genetics Selection Evolution* **2016**, 48, 52, 10.1186/s12711-016-0229-6
Seven goat breeds from Pakistan	50K Illumina BeadChip	Animal Genome Databases, https://www.animalgenome.org/repository/pub/CAAS2017.0629/	Kumar, C.; Song, S.; Dewani, P.; Kumar, M.; Parkash, O.; Ma, Y.; Malhi, K. K.; Yang, N.; Mwacharo, J. M.; He, X.; Jiang, L. Population structure, genetic diversity and selection signatures within seven indigenous Pakistani goat populations. *Animal genetics* **2018**, 49, 592–604, doi: 10.1111/age.12722
15 Chinese chicken populations	600K Affymetrix Axiom Chicken Genotyping Array	https://figshare.com/s/efe1bbbf60078f2f08aa	Zhang, J.; Nie, C.; Li, X.; Ning, Z.; Chen, Y.; Jia, Y.; Han, J.; Wang, L.; Lv, X.; Yang, W.; Qu, L. Genome-Wide Population Genetic Analysis of Commercial, Indigenous, Game, and Wild Chickens Using 600K SNP Microarray Data. *Frontiers in genetics* **2020**, 11, doi: 10.3389/fgene.2020.543294
Three Chinese indigenous chicken breeds	WGS	ENA, accession number PRJEB27583	Zhang, M.; Han, W.; Tang, H.; Li, G.; Zhang, M.; Xu, R.; Liu, Y.; Yang, T.; Li, W.; Zou, J.; Wu, K. *BMC genomics* **2018**, 19, 598, doi:10.1186/s12864-018-4973-6

[1]European Nucleotide Archive [2]Whole genome sequencing
